# “We Always Hurt the Things We Love”—Unnoticed Abuse of Companion Animals

**DOI:** 10.3390/ani8090157

**Published:** 2018-09-18

**Authors:** Bernard E. Rollin

**Affiliations:** Departments of Philosophy, Biomedical Sciences and Animal Sciences, Colorado State University, Fort Collins, CO 80523, USA; Bernard.Rollin@colostate.edu

**Keywords:** dogs as family, genetic diseases, genetic diseases and breed standards, prevalence of diseases in popular breeds

## Abstract

**Simple Summary:**

Over the last half-century, the role of companion animals in human society has changed dramatically. Where these animals were once seen as replaceable trinkets, they have now come to be viewed as “members of the family” by the majority of people who live with companion animals. Yet, despite this new status, we continue to inflict significant harm on these animals, and what is most tragic is that few animal owners take cognizance of the avoidable suffering which these animals go through. What I am primarily referring to is the proliferation of genetic diseases that characterizes pedigreed breeds. Most people bring dogs home while knowing nothing of their biology and genetics. Furthermore, many of the breed standards serve to augment, rather than minimize, these genetic problems that lead to a low quality of life and are also life-threatening. In general, the most popular breeds suffer the greatest number of genetic problems. These problems are perpetuated by non-rational considerations, such as trendiness, appearance in movies, and suchlike.

**Abstract:**

Despite the fact that companion animals enjoy the status of “members of the family” in contemporary society, there are numerous diseases affecting the longevity of these animals and their quality of life. Some of the most pervasive and damaging problems accrue to pedigreed animals whose genetic lines contain many major and severe diseases which are detrimental to both the quality and length of life. If one considers the most popular dog breeds in the United States, the top 10 include the Labrador Retriever, German Shepherd, Golden Retriever, French Bulldog, Beagle, Poodle, Rottweiler, Yorkshire Terrier, and German Shorthaired Pointer. Some idea of the pervasiveness of genetic defects across breeds can be gleaned from a recent book detailing genetic predisposition to disease. The book contains 93 pages of references. The list of diseases for the most popular dog, the Labrador Retriever, is 6.25 pages long. Yet, despite the tragic consequences of such diseases in animals regarded as beloved family members, breed standards associated with these diseases remain unchanged. This represents a major tragedy to which insufficient attention is paid. The point of this paper is to show that even as dogs have increasingly become viewed as “members of the family”, this status is belied by the proliferation of genetic diseases perpetuated by breed standards.

No one with any sense of cultural change can fail to notice the elevated status of companion animals in our society. Indeed, the very use of the term “companion animals” as opposed to “pets” eloquently bespeaks the way in which the augmented status has entered into the language of political correctness.

Illustrative of this change is an event that occurred when I was a teenager more than 50 years ago and living in Brooklyn, New York. I was standing outside my house when one of my neighbors, a woman in her mid-50s, suddenly began running down the middle of our quiet street, wearing a bathrobe and weeping hysterically while holding the body of her dead Chihuahua. She was screaming over and over, “First my mother, and now you!”.

Her unashamed and very public display quickly drew a crowd. The dominant response was, “For Christ’s sake, it’s just a dog; you can get another one!” Manifestly, the expression of that sentiment was of no consolation to her. In fact, the lack of evidenced empathy and understanding simply brought forth a fresh bout of tears.

No person today possessed of even minimal sensitivity would respond the way the neighbors did [1]. According to repeated surveys, a minimum of 89% of dog owners and up to a maximum of 99% profess to viewing their animal companions as “members of the family” Whereas dogs with hind end weakness would have once been euthanized, today one can purchase carts that essentially replace the hind end and allow mobility for the animal. Many people with geriatric animals will not even go on vacation if it means leaving the animal in a kennel. “Doggy day care” is a growth industry, as are luxurious (and expensive) boarding kennels. More than 20 years ago, the *New Yorker* reflected the new status of dogs in a cartoon, showing a wedding reception line with the dog shaking hands with everyone. One guest is saying to another something like “I know it’s odd, but they say he is a member of the family”.

I recall suffering from severe asthma occasioned by horribly polluted air in Manhattan. As any asthmatic knows, the effect of asthma is potentiated by anxiety and stress. In my case, the source of that stress was, in large part, the PhD program in philosophy at Columbia University in which I was enrolled. Virtually all doctoral programs at Columbia were extremely impersonal to say the least, and graduate students were seen as expendable and easily replaceable. My major sources of comfort were my dog and my cat, who not only provided me with much-needed affection, but in the case of the dog walking with my dog was also my sole source of exercise which helped mitigate the asthma.

When the condition became intolerable and I was visiting the emergency room multiple times per week, I made an appointment with the Chief of Allergy at the Columbia Medical School. After I explained my situation, he proceeded to put me on steroids for multiple years (which gravely compromised my health) and insisted that I “get rid of the dog and cat.” His total insensitivity to the nuances of my condition left me with an enduring distrust of the medical profession. (As it happened, leaving Columbia and New York City to move to Colorado completely mitigated my asthmatic condition, and within six months I was running).

A striking example of the new status of companion animals, particularly dogs, traces back to the early 1980s with the creation of the Animal Cancer Center at Colorado State University. At that time, a front-page story in the Wall Street Journal reported that there were clients at the cancer center spending more than six figures on treating their animals’ cancers, including one business executive who camped in the veterinary hospital parking lot in his RV for the entire six-week duration of the cancer treatment. The proliferation of specialty practices in veterinary medicine eloquently attests to the same point.

One can conjecture with some plausibility why this societal change in attitude has occurred: We are societally isolated from one another, particularly in urban and suburban locales. *Gesellschaft*, i.e., disconnected aggregates of individuals, has ever-increasingly replaced *gemeinschaft*, i.e., tightly bonded organic social unity. Divorce affects slightly less than half of marriages, leading to a feeling that home is no longer a stable source of comfort and belonging, particularly among children. (I often ask students in my introductory courses how many of them come from “broken homes”, and the rate closely mirrors the societal situation. I used to ask those students if it hurts, but stopped doing so when many of them started to cry). Furthermore, career opportunities are often nonexistent in small communities, so young people are drawn to urban contexts, thereby eroding the communal sense present in such communities, and also eroding extended families. As a person who spent the first 25 years of his life in New York City, I can attest to how difficult it is to make friends, particularly for young women who inevitably attract “circling predators”.

In fact, one of the few legitimate reasons for talking to and interacting with strangers in New York City is if one is pushing a baby carriage or walking a dog. Communities of people taking their dog to parks are able to form groups at the same time, creating significant camaraderie, and I was part of such a community. Even though we didn’t know each other’s names, and in many cases just referred to each other as “Fido’s person” or “Red’s owner,” we cared for each other. There was one time when the owner of a giant German Shepherd required hospitalization for a week. During that period, many of us took turns taking the dog to the park. New York paranoia was thrown to the winds, and we shared a key to the owner’s apartment.

A few years later, I remarked to Phil, the owner of the German Shepherd, that my physician was threatening to put me in hospital because of my recurrent asthma. The next day at the park, Phil handed me an envelope. I looked inside, and found a key. When I inquired him about it, he replied, “It is a key to my cabin in Thunder Bay, Ontario in the country. Go there and breathe”. That moment beautifully exemplified the extent to which a dog can serve to break down barriers and create bonds among people.

Perhaps the reader can now comprehend why I was so horrified to have the allergy specialist tell me to get rid of the dog and the cat. In any case, loneliness, social isolation and friendlessness are rife in today’s society, and companion animals are perhaps the best antidote. I asked the students in my class of 30 a few weeks ago, how many of them (average age about 25) had ever been betrayed by a friend, a lover, or relative. Every hand went up! I then asked them how many had been betrayed by an animal. No hands went up! In a sense, that says it all.

I could continue in this vein indefinitely, talking about dogs as an effective way of ameliorating PTSD, or dogs utilized in hospitals to dispel the fear, anxiety, and pain (yes pain!) associated with being hospitalized, or dogs being used to alleviate anxiety, and of course for more obvious services to people, such as working with the blind, as well as less obvious services. One of my blind friends who was tragically blinded by a psychopath putting acid in bottles of eye-drops had a seeing-eye dog named Hedda Steam. She told me that had it not been for Hedda, she would have seriously contemplated suicide.

Dogs are used to reduce stress in people experiencing difficult situations, ranging from being stuck in airports to consoling students in universities when they are first away from home. Less obvious services include sniffing out bombs in public places, finding lost people, anticipating epileptic seizures and protecting human epileptic patients from falls during seizures, detecting tumors in humans, and a recent story I read was of a shelter dog, newly adopted by an Australian couple, who interposed his body between a venomous snake and an infant, took the bite in place of the infant, and killed the snake.

Commensurate with this new status of companion animals has been something of a decline in immorality perpetrated on them. There are, for example, fewer animals euthanized in shelters per year. According to the website of the American Society for the Prevention of Cruelty to Animals, the number of dogs and cats euthanized has dropped from 2.6 million in 2011 to 1.5 million currently [2].

More people today are finding creative ways to use animals from shelters, such as many kinds of service dogs. Advocacy groups such as the Beagle Freedom Project devote their attention to finding homes for animals that have been used in research but can now be adopted out.

In other ways, however, we seem to have made little progress. A significant percentage of animals still come from puppy mills, pet stores, and flea markets. To be fair, attempts are being made to set standards for puppy mills, for example by a task force chartered by both the pet industry and national humane organizations on which I served. Some jurisdictions have banned pet stores based on the realization that many of the animals sold therein come from puppy mills and have been produced under execrable conditions. Most egregious is the fact that, as we shall shortly discuss, we choose our companion animals in ways that virtually guarantee irremediable defects and a miserable life.

In the face of the bounty these animals bestow upon us, simple reciprocity and justice would demand that we do the same for them. And in some ways, we do. For example, as mentioned previously, the numbers of animals killed in “shelters” has declined significantly, although we are still killing millions per year. Sadistic “training methods” and devices such as shock collars are still commonly used and readily available for purchase, with no questions asked. I found 23,000,000 references on Google. Ironically, the use of shock can backfire with the wrong behavior being reinforced. In addition, as B.F. Skinner once told me in a letter, he gave up negative reinforcement for training early in his career because positive reinforcement works far better.

Let us recall, as mentioned earlier, that for a significant number of dog owners, the animals are perceived as “members of the family”. They are objects of love and ever-increasingly become the central foci of our attention, where in many cases they are helping to indeed bond the family together when all participate in playing with or exercising the animals.

Performing a thought experiment will help to illuminate the degree of atrocity that we perpetrate on dogs. Imagine that one could freely choose one’s children in terms of various phenotypic traits, physical and psychological, in accordance with one’s values and aesthetic predilections. That means we could decide in advance whether the child was tall or short, blonde or brunette, slight or muscular, physically beautiful or average looking, saintly or aggressive, possessed of musical ability or mathematical proficiency. In fact, all possible phenotypic traits that would be of interest to a parent. Let us further suppose that there was a cost of some significance to the child in terms of health, disease, and general quality of life if children were thus chosen. In other words, choosing a certain phenotype would create a high degree of certainty that the child would acquire cancer (as in the BRC gene), be cognitively deficient, physically impaired, or have compromised ability to see, hear, touch, taste, smell, reason, or locomote, and myriad other defects and diseases.

Only a demented or psychopathic person would choose their child’s traits in ways that would or even could harm the child in the future, or compromise their abilities and their quality of life. In fact, most of us shudder at the very thought of such a world. Yet we proceed to demonstrate that it is precisely this very way that we choose the traits of the dogs we profess to love and cherish. The fact that this is done not out of malice but out of desire for trendiness or ignorance does not, as we will see, mitigate the horror of what we do. In fact, any time we choose an animal on the basis of how it looks, what it could win in dog shows, or what has recently appeared in a popular movie, we are doing precisely what is described above. As I have argued elsewhere, too many people acquiring dogs view the animals as trophies, as something to enhance the owner’s ego, as if they were specialty cars at car shows. Having been to numerous dog shows, I am well-aware of this phenomenon, beautifully documented in the superb comedy *Best in Show*.

Consider the most popular dog breeds in the United States. According to the American Kennel Club, the number-one most popular breed in 2018 is the Labrador Retriever, second is the German Shepherd, third are Golden Retrievers, fourth are French Bulldogs, and fifth is the Bulldog [3]. Sixth through 10th are the Beagle, Poodle, Rottweiler, Yorkshire Terrier, and the German Shorthaired Pointer. The full list comprises 190 breeds, but an examination of the top 10 will suffice to make our point.

A recent and superb book authored by three British veterinarians who are experts in canine genetics is devoted to, as the title indicates, *Breed Dispositions to Disease in Dogs and Cats* [4]. Anyone and everyone even considering acquiring a purebred dog should consult this invaluable, evidence-based volume to avoid heartache and aggravation later down the road. The authors are very serious about being science- and evidence-based in their propositions—there is an unbelievable 93-page reference list at the end of the book, and even more remarkable is the 240 pages of small print listing the diseases affecting purebreds.

Consistent with the authors’ avowed commitment to work strictly from science, they do not remark on the ethical issues occasioned by the depressing list of diseases. On the other hand, the list speaks for itself, especially when one reads the descriptions of the diseases in the second part of the book.

In order to illustrate what we are talking about in a very direct and dramatic way, it is worth examining the diseases genetically perpetuated in some of the top dog breeds. As just mentioned, the Labrador Retriever is the number-one most popular breed. Amazingly, the book of genetic diseases lists well over 60 diseases for which Labradors are at risk. These include cardiovascular conditions such as cardiac arrhythmia, atrioventricular block, pericardial effusion, and tricuspid valve dysplasia. Endocrine conditions include diabetes and hypothyroidism. Gastrointestinal conditions include chronic hepatitis, congenital portosystemic shunt, and esophageal infection. Hematological and immunological conditions include Hemophilia A and B, and selective IGA deficiency associated with “recurrent infections of mucosal sites and increased susceptibility to immune mediated disease” [5]. Infectious conditions include blastomycosis and Lyme disease, which is a tick-borne disease caused by a spirochete bacterium of the *Borrelia burgdorferi* group. In areas where the disease is endemic, up to 50% of dogs may harbor the pathogen, but only some exhibit symptoms. These include Labrador Retrievers and Golden Retrievers who are genetically predisposed to the condition. In some cases, Lyme disease causes lameness and severe kidney problems. Metabolic conditions can also occur, such as obesity, and musculoskeletal conditions are particularly prevalent. These include cranial cruciate ligament disease, discospondylitis, elbow dysplasia, hip dysplasia, limber tail, lumbosacral transitional vertebrae, metaphyseal osteopathy, muscular stiffness, several types of myopathy, osteochondrosis of the elbow, osteochondrosis of the shoulder, osteochondrosis of the stifle, osteochondrosis of the tarsus, panosteitis (intermittent lameness), patellar luxation, and polyarthritis.

Moving on to neoplastic disease (i.e., cancer), one encounters a significantly depressing litany. Here is a list of cancers to which Labradors are prone: Cutaneous hemangioma; Hemangiosarcoma; Histiocytic sarcoma complex; Infiltrative lipoma; Mast cell tumor; Melanoma; Osteosarcoma; Sarcoma of soft tissue; Squamous cell carcinoma of digits; Digital tumors and Thymoma.

Neurological conditions include: exercise-induced collapse; idiopathic epilepsy; leucodystrophy; narcolepsy–cataplexy; paroxysmal dyskinesia.

Ocular conditions include: cataracts; entropion; histiocytic ocular sarcoma; mast cell tumor-conjunctival; oculoskeletal dysplasia; progressive retinal atrophy; retinal pigment epithelial dystrophy; uveal cysts.

Renal and urinary conditions include ectopic ureter and Lyme nephritis, and finally, to round out the list, is laryngeal paralysis–polyneuropathy syndrome.

When I first considered writing this paper, I grossly underestimated in my own mind the prevalence of genetic disease predisposition in the top 10 most popular dogs. As I did the research, however, it became clear to me that such a list would be far too ponderous and, in fact, unreadable. The reader should be assured that, to varying degrees, the sort of diseases listed for the Labrador would comprise similar lists for the other breeds. In the case of the German Shepherd, the second most popular breed, the book on genetic diseases lists more than 80 afflictions!

While it may be tempting to elaborate on the genetic mechanisms which create the sorts of problems we have enumerated, a very rudimentary, high-school-level grasp of breeding is all that is needed. When very large groups of diverse individuals are used for breeding, one generally minimizes the chance of deleterious recessive genes combining to create defective individuals. Though numerous animals may carry the genes in question, they are less likely to determine the phenotype of the animal achieved by reproduction, since both parents must carry and contribute the harmful recessive gene to create phenotypic (i.e., actual, observable, functional) defects. On the other hand, when the breeding pool is very small, the chances of both parents contributing a negative recessive gene is greatly amplified, thereby enhancing the possibility of genetically and functionally defective offspring [5].

This is precisely what occurs in inbred populations of humans, who display such traits as six fingers or intellectual disability by virtue of each parent contributing a defective gene. It is also the way hemophilia became a serious problem in European royal families, given the practice of mating only with other royal family members. This is of course a major rational justification for the ancient taboo against incest.

In animal breeding, on the other hand, it is very common to create “incestuous crosses” to highlight and disseminate phenotypes that are considered highly desirable. The price, of course, is the proliferation of diseases of the sort enumerated in the book we have alluded to.

Anyone who has bought or adopted one or more purebred dogs has likely suffered from the heartache of seeing them die prematurely, and often horribly, of the genetic diseases so perpetuated. My beloved Great Dane, discussed earlier, died of osteosarcoma or bone cancer at a relatively young age after undergoing radical procedures, including a foreleg amputation which did not forestall the cancer. So sweet a dog was she that when the surgeon/oncologist had to euthanize her, despite his being a tough cowboy, he cried along with me. I had similar experiences with a German Shepherd who was paralyzed by degenerative spinal mylopathy, a very common disease of German Shepherds, again dying unpleasantly before his time. That is why people who know dogs advise adopting a mixed breed. Of course, there is no guarantee that a mixed breed will not suffer from genetic problems, but nonetheless the odds will be far more in your favor.

No one can ever forget the death of a dog. As humorist Dorothy Parker once remarked, “The only thing wrong with dogs is that they die too soon”. (Recall our earlier anecdote regarding the woman whose Chihuahua died.) The normal lifespan of a dog should be 10–15 years. Large breeds live for about 8 years. As in the case with genetic and other horrendous diseases, one is often forced to euthanize the animal in great suffering to curtail the pain and loss of quality of life. Even though euthanasia is medically required in many cases, no one should underestimate the degree of trauma caused by, as one animal owner said to me, “killing my best friend”. Choosing death for such a being is no small trauma.

No person who has lived through euthanizing a close animal companion can ever forget it. There is something horrendous about watching a friend draw their last breath, and one wonders forever whether they chose euthanasia at the right time. In retrospect, one very often feels guilty that they waited too long or, alternatively, that they acted too quickly. A sensitive veterinarian who knows the dog and your relationship with the animal can help immeasurably in easing an owner’s pain and guilt.

There is no reason that why genetic disease occurring on the basis of current breed standards should continue. As is the case with so much animal abuse and so many animal welfare issues, the key to a proper solution lies with the consumer. If people would stop choosing companion animals just because they are “cute”, the new trend, featured in a film or TV program, or because they want one to compete in dog shows, supply would follow demand. Pugs are undeniably cute, but also suffer from dystocia—in Britain, over 27% of pugs did not experience a normal birth but required cesarean section [4]. Pugs are also afflicted with Brachycephalic Destructive Airway Syndrome (BOAS)—50% of U.S. BOAS caseloads were pugs [4]. This condition also affects the very popular French Bulldog, and numerous other flat-faced dogs and cats. There are few problems as devastating to animals and animal owners as watching their animals struggle to breathe. Bulldogs, again among the top 10 in popularity, also suffer from the above set of problems, and are, ironically, as far removed as an animal could be from their highly functional predecessors hundreds of years ago. So severe is the problem that the British Veterinary Association constituted a working group on BOAS. The disease requires major surgical intervention that can cost $1500 or more.

Prospective dog owners would be wise to consult with veterinarians on the potential health problems of the breed they are considering. According to an article on this issue, a recent study indicated that 48% of companion animal veterinarians were advising clients against purchasing a pedigree dog breed due to inherited disorders [6]. Personally, I have strongly advised friends who wish to acquire a dog to visit a local or regional shelter and choose a mixed breed. Not only is one diminishing the chances of caring for a dog with a genetic disease, but one is also saving an innocent life. (No one has ever come back to me complaining about my advice.) Even more importantly, you will avoid falling into the trap of hurting something that you love and that loves you wholeheartedly and unequivocally. What we need is healthy and happy dogs, not, as a veterinarian expert on genetic diseases once said to me, “living statues”.
